# Obstructive Sleep Apnea: characterization of the obstructive site and type of collapse

**DOI:** 10.1590/2317-1782/20212021208

**Published:** 2022-05-16

**Authors:** Andresa Santos da Silva, Fabio Augusto Winckler Rabelo, Eric Thuler, Fabiane Kayamori, Esther Mandelbaum Gonçalves Bianchini

**Affiliations:** 1 Programa de Pós-graduação em Fonoaudiologia, Pontifícia Universidade Católica de São Paulo – PUCSP - São Paulo (SP), Brasil.; 2 Departamento de Otorrinolaringologia, Hospital Samaritano - São Paulo (SP), Brasil.; 3 Departamento de Otorrinolaringologia, Hospital Sírio Libanês – HSL - São Paulo (SP), Brasil.

**Keywords:** Obstructive Sleep Apnea, Snoring, Pharynx, Endoscopy, Myofunctional Therapy, Speech Language and Hearing Sciences

## Abstract

Obstructive Sleep Apnea is characterized by recurrent episodes of partial or complete collapse of the pharynx, followed by decreased oxyhemoglobin saturation and frequent arousals. It is regarded as a public health issue with important night and day symptoms that impact life quality. Its effects are associated with the areas of competence of Speech and Language Pathologists. To establish efficient diagnosis and treatment methods, professionals must know the pathogenesis of upper airway obstruction during sleep. This study seeks to enlarge the understanding of obstructive sleep apnea pathophysiology, eligibility of individualized therapeutic procedures and guidance for orofacial myofunctional therapy by describing and illustrating the locations and types of upper airway collapse during sleep. We analyzed original records of Drug Induced Sleep Endoscopy exams of a series of cases with polysomnographic diagnosis of obstructive sleep apnea following the proper ethical processes. The images of the exam recordings were analyzed by five professionals with expertise in the sleep area. Obstructive sites and types of collapse were presented according to the current classification. The videos were divided into screenshots, originating figures from each anatomical site: without collapse and collapsed. The results are visualized in the images of the cases showing a predominance of velopharyngeal collapse: anteroposterior, lateral, or concentric; oropharyngeal lateral collapse; tongue anteroposterior collapse and anteroposterior collapse of the epiglottis. Understanding the obstruction sites and types of collapse illustrated in this study may help to predict therapeutic responses and learn the limitations or direct individual proposals patient.

## INTRODUCTION

Obstructive Sleep Apnea (OSA) is characterized by recurrent episodes of partial or complete pharyngeal collapse, causing hypopnea or apnea events, respectively, which may be followed by decreased oxyhemoglobin saturation and/or frequent arousals. It is considered a public health issue with important nocturnal and daytime symptoms that impact life quality^(1)^. Common symptoms include excessive daytime sleepiness, lack of attention and memory, mood change, increased risk of accidents, and long-term cardiometabolic diseases^(1)^. When left untreated, the impacts of sleep-disordered breathing (SDB) can also have effects such as neurobehavioral and cognitive deficits, as well as changes in voice quality, swallowing, auditory behavior, and speech fluency interfering with different levels of communication throughout childhood, adolescence, adulthood, and senility^(1)^. These effects are strongly associated with the areas of competence of the Speech-Language Pathologists.

The pathogenesis of OSA is multifactorial and is associated with anatomical and neuromuscular factors. Regarding anatomical factors, studies have demonstrated^(2,3)^ the role of craniofacial structures, such as maxillary deficiency, high and narrow hard palate, and reduced mandible. The following neuromuscular factors stand out: narrow or collapsible pharynx and hypertrophied soft tissues, such as enlarged palatine tonsils and pharyngeal pillars, as well as size, shape, volume of the soft palate, and lingual tonsils. Aging and obesity are also considered contributing factors, respectively, for reduced tone and permeability of the Upper Airway (UA). Decreased muscle tone and hypomobility contribute to increased volume and sagging of the soft tissue structure, which can lead to upper airway collapse, micro-arousals, and sleep fragmentation^(4)^.

The standard treatment for OSA is the use of a positive airway pressure (PAP) device during sleep^(2)^; however, poor adherence to this treatment compromises its effectiveness. The use of mandibular advancement devices (MAD), with better acceptance, is also an effective clinical therapy aimed at increasing the diameter of the upper airway with titratable mandibular advancement^(3)^. Surgical treatment for OSA includes soft tissue procedures of the oropharynx and base of the tongue, as well as skeletal surgery, where maxillary expansion and maxillomandibular advancement are also effective options^(5)^, albeit invasive.

Orofacial Myofunctional Therapy (OMT) is introduced as an alternative and/or complementary clinical treatment option for sleep-disordered breathing (SDB). The OMT proposes to promote changes in the oropharyngeal and functional muscles by means of isotonic and isometric exercises using the orofacial and oropharyngeal muscles, in addition to the oropharyngeal functional organization^(6)^. This set of techniques and procedures has shown satisfactory results in treating OSA, with significant improvement in early symptoms and better life quality^(7)^. However, not all individuals with OSA are eligible for OMT^(7,8)^ and it is crucial to define the correct diagnoses for patient selection.

Type 1 polysomnography (PSG1) is the gold standard in the diagnostic investigation of sleep disorders (SD) and consists of the simultaneous recording of physiological variables during sleep that indicates the distribution of sleep stages, occurrence and characterization of central and/or obstructive respiratory events, blood gas monitoring such as oxygen saturation and carbon dioxide concentration, in addition to electromyogram and movement sensors differentiating various interferences that can define micro-arousals and consequent poor sleep quality. PSG1 is valuable in the diagnosis of SD in general since its symptoms can be related not only to the occurrence of SDB. It is also a decisive assessment for the detection, characterization, and severity of OSA, however, it does not identify neither the obstructive site nor the type of collapse^(8)^.

Determining the obstructive site of the UA and the pattern of collapse is decisive to understand the problem and guide its treatment, whether surgical or clinical^(9)^. Drug-induced sleep endoscopy (DISE) is an endoscopic assessment of the UA during medication-induced sleep. It consists of a three-dimensional visualization of the UA that allows real-time assessment of obstructive sites under dynamic conditions^(9,10)^. Studies have shown that the obstruction sites and types of collapse may define the results of different treatments, suggesting that detecting the obstructive site and understanding the mechanisms that favor its occurrence influence the therapeutic decision^(8-11)^.

Considering that characterizing the obstructive site and understanding the collapse pattern in patients with SDB provide specific and individualized directions aimed at promoting more promising results, it is fundamental that Speech-Language Pathologists become aware of these data and investigate specific OMT procedures for collapse sites and types.

Thus, aiming to enlarging the understanding of the OSA pathophysiology, the eligibility of individualized therapeutic procedures and guidance for orofacial myofunctional therapy, the goal of this study is to describe and illustrate the sites and types of upper airway collapse during sleep according to data from the DISE.

## PRESENTATION OF CLINICAL CASES

This is an exploratory-descriptive case study approved by the Ethics and Research Committee of “Pontifícia Universidade Católica de São Paulo” (PUC-SP), protocol number 1.964.298 and CAEE 63408516.8.0000.5482.

We collected the data from 25 consecutive patients at the Otorhinolaryngology outpatient clinic of the Hospital Samaritano de São Paulo, according to the proper ethical procedures, with a polysomnographic diagnosis of OSA and indication of DISE, for therapeutic definition. This is an elective assessment defined and carried out by the otolaryngologist (ENT), responsible for the case. The exams were performed at the endoscopy center using a flexible Olympus® / Scad® 3.4mm nasofibrolaryngoscope supervised by an anesthesiologist under controlled target infusion of propofol-induced sleep, according to the aforementioned protocol^(12)^ capable of reproducing the same Apnea and Hypopnea Index (AHI) as natural sleep. Cardiorespiratory parameters were monitored, and all safety equipment was available to respond to any possible emergency. All exams were monitored by a Speech-Language Pathologist qualified in the area of ​​sleep and the images were digitally recorded for further analysis.

The selection process of the DISE exam videos followed an inclusion criterion for selecting the images, allowing to visualize each possible location and types of collapse presented.

Patients with a body mass index (BMI) >29.9 and comorbidities, as well as those whose samples implicated a technical issue compromising the quality of the video were excluded.

Based on these criteria, 11 cases were selected with a mean age of 41 years, minimum of 28 years and maximum of 45 years; mean BMI of 25.05, minimum of 23.4 and maximum of 27.3; all male individuals with moderate OSA, thus reflecting the findings in this study.

The participants of this research are part of a larger research program and signed the Free and Informed Consent Term, thus consenting to the dissemination of their results.

### Sites and types of UA collapse

Upper airway obstruction sites were described according to the “VOTE”^(9,10)^ classification: (V) Velopharyngeal, involving the soft palate, uvula and tissue of the lateral wall of the rhino pharynx; (O) Oropharyngeal, involving soft tissues of the lateral wall of the oropharynx and palatine tonsils; (T) Tongue (lingual), involving the base of the tongue and the posterior wall of the oropharynx; (E) Epiglottic, involving the epiglottic cartilage, aryepiglottic fold, and supraglottic area that may collapse due to reduced structural rigidity of the cartilage or its posterior displacement against the posterior wall of the pharynx. Each of the sites is also classified according to their respective types of collapse: anteroposterior, lateral, or concentric. The clogging degrees are classified as follows: (0) no significant clogging <50%, no vibration; (1) partial obstruction (50 to 75%) with vibration; (2) complete obstruction (> 75%), and (x) not visible.

The images were selected and analyzed by the authors of this study, two otolaryngologists and three Speech-Language Pathologists with expertise in the area of ​​sleep, followed by joint confirmation according to the location and type of collapse presented.

To facilitate the presentation and visualization of the collapse sites, as well as the analysis of each case, screenshots were taken at two or three moments of each site: without collapse, moment of transition, and collapse. However, not all cases were illustrated with images of the transition moment as a function of the rapidity of the collapse occurrence, due to its variability. The cases in which the speed of collapse allowed capturing the transition moment were presented as the intermediate image.

The obstruction degree selected was preferably complete or partial. The degrees depicting vibration were not exposed, as the screenshots do not show the vibration and dynamic images are only observable in video. While the images have been turned into photos to allow for publication, video recordings are available.

Chart 1 illustrates how the images were viewed and interpreted, representing the view of the nasofibrolaryngoscope positioned respectively in the velopharynx and oropharynx, without collapse.

**Chart 1:** Example of the two positions of the nasofibroscope for analysis of the images obtained: velopharynx and oropharynx

**Nasofibrolaryngoscopic images with the individual awake, at rest.**		
Nasofibrolaryngoscope positioned in the velopharynx	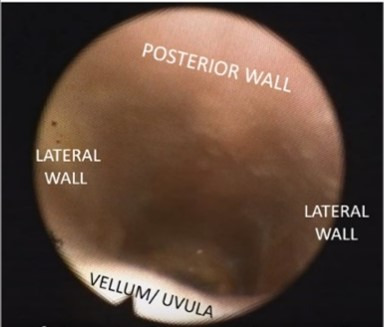	Subject awake, lying down, breathing through his nose
Nasofibrolaryngoscope positioned in the oropharynx	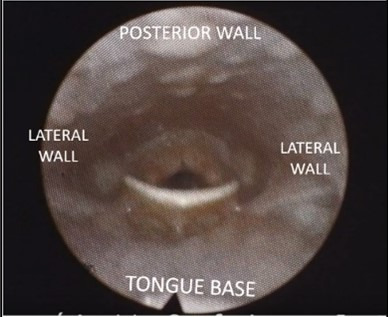	Subject awake, lying down, breathing through his nose

Table 1 presents the data regarding the BMI, location, and type of collapse of each patient analyzed. A larger number of velopharyngeal collapses was observed (five cases), followed by lingual collapse (three cases), oropharyngeal collapse (two cases), and epiglottic collapse (two cases). Note that only one case had two types of collapse: complete lingual and epiglottic.

**Table 1:** Data and findings of the participating subjects

**Subjects**	**BMI**	**Collapse site**	**Collapse type**
1	23.4	V: Velopharyngeal Site	Complete anteroposterior
2	25.2	V: Velopharyngeal Site	Complete anteroposterior
3	24.3	V: Velopharyngeal Site	Full circumferential
4	27.3	V: Velopharyngeal Site	Full lateral
5	24.8	V: Velopharyngeal Site	Full circumferential
6	24.5	O: Oropharyngeal site	Full lateral
7	26.2	O: Oropharyngeal site	Partial lateral
8	26.3	T: Tongue (Lingual) Site	Complete anteroposterior in hypopharynx with lingual tonsillar component
9	25.4	T: Tongue (Lingual) Site	Complete anteroposterior in the hypopharynx with minimal tonsillar component
10	24.5	E: Epiglottic site	Complete anteroposterior
11	23.7	T: Lingual Site	Complete anteroposterior in the hypopharynx without lingual tonsillar component

The illustrations of the cases were presented according to the location and type of collapse, described below.

- Velopharyngeal site

**Figure 1:**
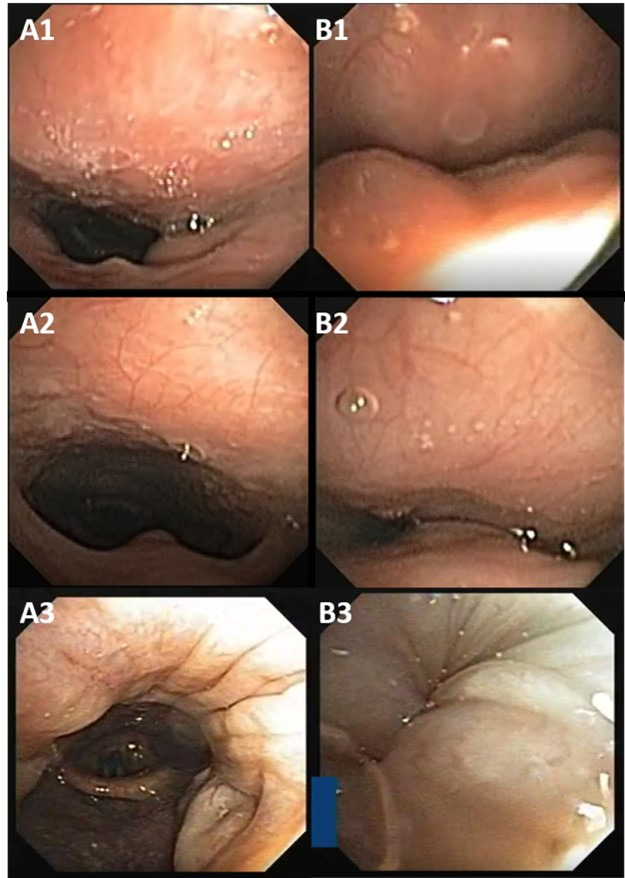
Velopharyngeal site (V). “A” images without collapse; “B” images during collapse

**Figure 2:**
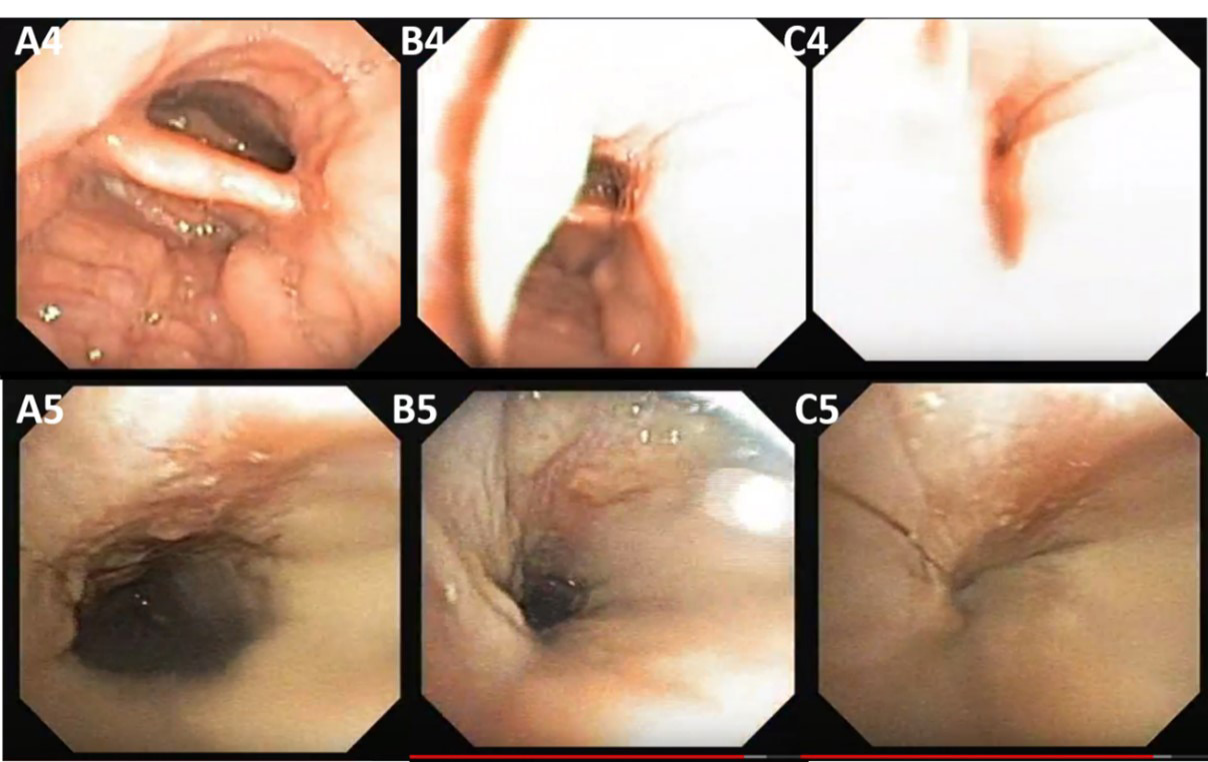
Velopharyngeal site (V). “A” images without collapse; ‘B’ images transition to collapse with partial constrictions, ‘C’ images during collapse

The velopharyngeal site can present three types of collapse: anteroposterior, lateral, or circumferential (concentric), as illustrated in Figures 1 and 2.

**Caption:** Subject 1: Image A1 without collapse, B1 with a complete anteroposterior collapse in the velopharynx

**Caption:** Subject 2: Image A2 without collapse, B2 with a complete anteroposterior collapse in the velopharynx

**Caption:** Subject 3: Image A3 without collapse, B3 with a complete circumferential collapse in the velopharynx

**Caption:** Subject 4: Image A4 without collapse, B4 transition to lateral collapse with partial constrictions, C4 complete lateral collapse in the velopharynx

**Caption:** Subject 5: Image A5 without collapse, B5 transition to circumferential collapse with partial constrictions, C5 complete circumferential collapse in the velopharynx

Anteroposterior collapse corresponds to constriction of the soft palate, uvula, and posterior wall of the velopharynx, as shown in Figure 1. Concentric or circumferential collapse corresponds to the simultaneous constriction of the soft palate, uvula, lateral wall, and posterior wall of the velopharynx, as illustrated in Figures 1 and 2. The lateral collapse corresponds to the constriction of the lateral walls of the rhinopharynx, as in Figure 2.

Subjects 1 and 2 had complete anteroposterior velopharyngeal collapse (Figure 1). Respectively A1 and A2 illustrate the velopharyngeal site without collapse, B1 and B2 exemplify complete anteroposterior collapse.

Subject 4 had complete lateral velopharyngeal collapse (Figure 2). Illustrated by captures at three different times, showing, respectively, A4 velopharyngeal site without collapse, B4 during the transition to collapse, and C4 with complete lateral collapse.

Subjects 3 and 5 had a complete circumferential velopharyngeal collapse. The images of subject 3 (Figure 1) represent two different moments: A3, showing the velopharyngeal site without collapse and B3, complete circumferential collapse in the velopharynx. The images of subject 5 (Figure 2) were captured at three moments, A5 without collapse, B5 during the transition to collapse and C5 when the complete circumferential collapse occurred.

- Oropharyngeal site

**Figure 3:**
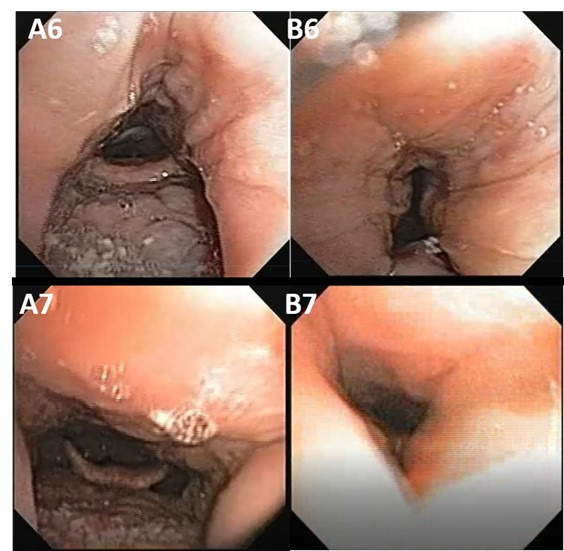
Oropharyngeal site (O). “A” images without collapse; “B” images with collapse

The oropharyngeal site may present one type of collapse: lateral, resulting from the constriction of the lateral walls of the oropharynx and depends not only on the size of the palatine tonsils, but also on the redundant soft tissue in this area, as shown in Figure 3, illustrated by the captures of subjects 6 and 7.

**Caption:** Subject 6: Image A6 without collapse, B6 with a complete collapse of the lateral walls of the oropharynx

**Caption:** Subject 7: Image A7 without collapse, B7 with partial collapse of the lateral walls of the oropharynx

The images were captured at two different times, without collapse A6 and A7 and when the lateral wall of the oropharynx B6 and B7 collapsed (Figure 3). It is noteworthy that both subjects had collapsed without enlarged palatine tonsils.

- Lingual site (hypopharynx)

**Figure 4:**
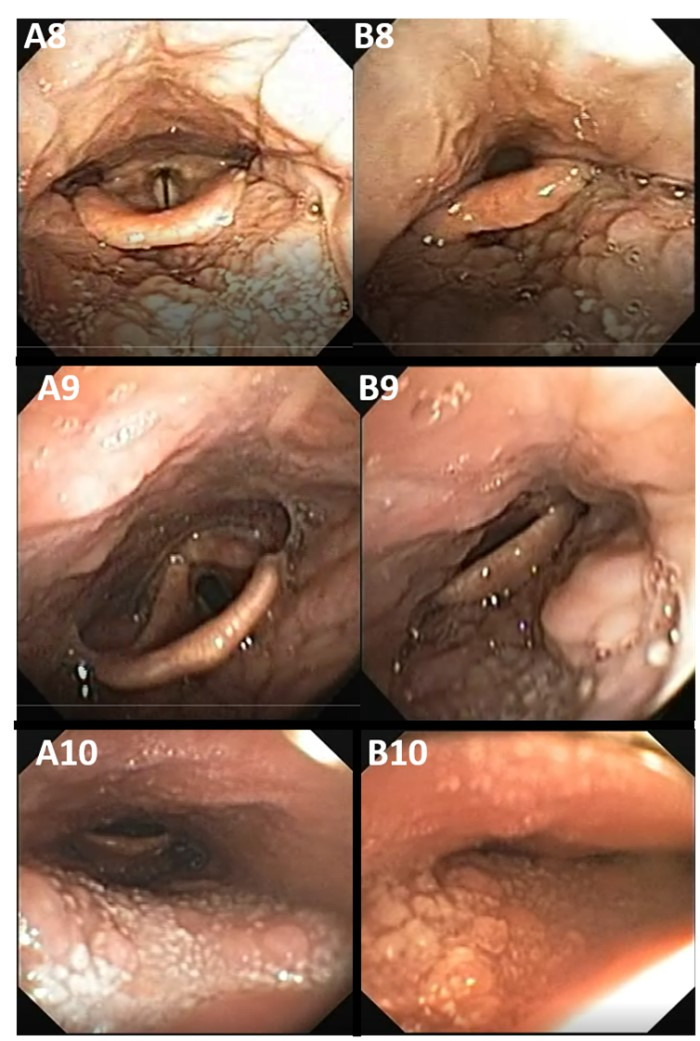
Site of the base of the tongue (T). “A” images without collapse; “B” images with collapse

The lingual site can present one type of collapse: anteroposterior, due to the constriction between the base of the tongue and the posterior wall of the hypopharynx, as illustrated in Figure 4, shown in the captures of the exams of subjects 8, 9, and 10.

**Caption:** Subject 8: Image A8 base of tongue with lingual tonsil hypertrophy without hypopharyngeal collapse; B8 with a complete anteroposterior collapse in the hypopharynx with a tonsillar component

**Caption:** Subject 9: Image A9 base of the tongue with minimal hypertrophy of the lingual tonsil without collapse in the hypopharynx; B9 with a complete anteroposterior collapse in the hypopharynx with minimal tonsillar component

**Caption:** Subject 10: Image A10 base of the tongue without hypertrophy of the lingual tonsil and collapse in the hypopharynx; B10 with a complete anteroposterior collapse in the hypopharynx without tonsillar component

Subject 8 has lingual tonsil hypertrophy and subject 9 has minimal unilateral hypertrophy of the tonsil. Captures A8 and A9 show the hypopharyngeal region without collapse to facilitate comparison with images B8 and B9, thus defining anteroposterior hypopharyngeal collapse with a lingual tonsillar component.

Subject 10 (A10 and B10) had anteroposterior collapse of the base of the tongue without any hypertrophy of the lingual tonsils. Capture A10 shows this site without collapse and B10 shows a complete anteroposterior collapse in the hypopharynx caused by posterior displacement of the base of the tongue after muscle relaxation. It is noteworthy that the three cases presented complete collapse, regardless of the volume of the lingual tonsil. However, this is considered an aggravating factor for this type of collapse.

- Epiglottic Site

**Figure 5:**
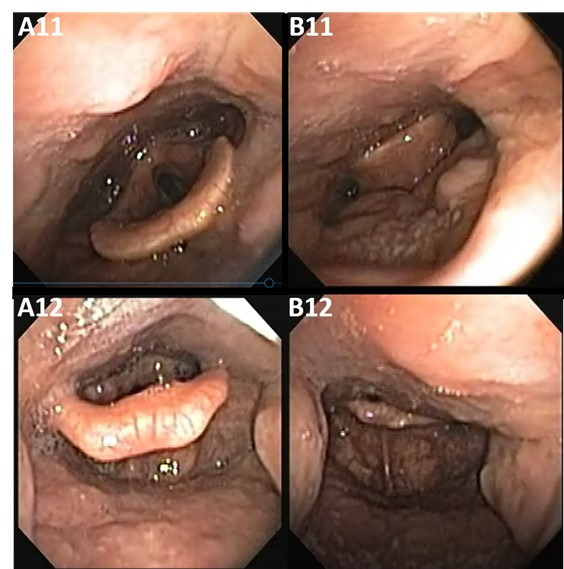
Epiglottic site (E). “A” images without collapse; “B” images with collapse

Regarding the epiglottis site, two types of collapse can be found: anteroposterior and lateral. In our sample, two cases of epiglottic collapse were found, subject 9 and subject 11, both anteroposterior type, as a consequence of the posterior displacement of the epiglottis against the posterior wall of the hypopharynx, as illustrated in Figure 5.

**Caption:** Subject 09: Image A11 epiglottic site without collapse, B11 complete anteroposterior collapse caused by posterior displacement of the epiglottis against the posterior hypopharyngeal wall with minimal lingual tonsillar component

**Caption:** Subject 11: Image A1: no collapse, B12 with complete anteroposterior collapse caused by posterior displacement of the epiglottis against the posterior hypopharyngeal wall, without lingual tonsillar component

Captures A11 and A12 demonstrate the site of the epiglottis without collapse, facilitating comparison with images B11 and B12, thus demonstrating the complete anteroposterior collapse of the epiglottis. It is noteworthy that subject 9 presented two types of collapse at different times of the DISE assessment: lingual (Figure 4) and epiglottic (Figure 5), both anteroposterior, suggesting a muscular component of tongue drop.

For this study, we found no cases that could illustrate the lateral collapse of the epiglottis site, which rarely involves instability of the epiglottis rigidity laterally^(10,13)^.

Among the limitations of the study, as it is an elective and high-cost assessment, we highlight the number of analyzed cases, which, although allowing to present almost all types of collapse, except lateral epiglottic collapse, could not allow to generalize the frequency. The absence of scientific evidence in the field of Speech-Language Pathology on the topic addressed so far hampers to establish comparisons with previous research or associations with a consequent broader discussion on the data presented.

## DISCUSSION

This study introduces a series of 11 cases aiming to describe and illustrate the sites and types of upper airway collapse during sleep and enlarge the understanding of the pathophysiology of OSA. A larger number of cases with collapse in the velopharyngeal site was found, followed by oropharyngeal collapse, lingual collapse, while epiglottic collapse was the least frequent. These data corroborate previous studies that demonstrate the velopharyngeal region as the most common site of upper airway collapse during sleep^(10,12,14)^. However, as this is a case series, data do not allow to generalize the frequency of occurrence of each site and type of collapse, nor the obstruction degree.

OSA has been widely studied in recent decades, several studies have enlarged the understanding of this disease, including its symptoms, diagnostic definition, and therapeutic approach. Although the use of positive airway pressure (PAP) devices has generated good results, poor adherence compromises their effectiveness, thus justifying the search for alternative treatment options^(2,3,5-7,11)^.

Regarding the speech-language pathology role in SDB^(6,7)^ there is evidence demonstrating that OMT, combined with oropharyngeal exercises and functional reorganization, is an effective treatment alternative for many patients. The therapy focuses on improving muscle competence, flexibility, and tone in the oropharyngeal region. Thus, it is important to seek instruments to optimize the understanding of sleep physiology and pathophysiology for a better definition for OMT eligibility in the treatment of SDB^(6,7,11)^.

DISE has been characterized as an important tool in detecting obstructive sites of the upper airway, assisting in a patient selection regarding the clinical and/or surgical therapeutic definition and respective results^(9,10,14)^. As for the clinical management aimed by the OMT, based on characterizing the obstructive site and type of collapse, potential therapeutic considerations should be addressed. The selection of exercises may be focused on those that act directly on the collapse region since it implies greater weakness in the muscles. Such clinical guidance contributes to the elaboration of individualized therapeutic planning focused on the affected musculature, not always verified in clinical assessment. The possibility of reducing the number of exercises proposed allows a better adherence to orofacial myofunctional treatment. Understanding the collapse should also consider a therapy that seeks to counter these collapses, that is, avoid reproducing the obstruction with a certain exercise and prioritize those that generate space opening, in addition to strengthening the affected structure. The exercises to be indicated can be those that do not provide the closing/obstruction movement. Based on these definitions, new controlled and randomized studies should be conducted to verify therapeutic results.

Comparing the types of collapse, a cohort study^(10)^ reports that patients who had a complete circumferential collapse in the velopharynx were significantly more likely to have a higher Apnea Hypopnea Index (AHI) and BMI than those who had anteroposterior collapse at the same obstructive site, who had a significantly lower BMI. In the case series herein, these data were not decisive since the BMI and the degree of OSA severity were similar. It is worth mentioning that studies indicate that circumferential collapse in the velopharynx is more difficult to treat than other sites and patterns of collapse, thus representing a more severe anatomical narrowing^10,13.^ The mechanical approach using a continuous positive pressure device, or even an intraoral device, is considered the most appropriate in these cases^(2,3)^. Therefore, the detection of complete circumferential collapse during DISE showed important therapeutic guidance^(14,15)^. In this sense, the OMT approach may not be sufficient to treat this type of collapse or may require greater definition in the choice of exercises depending on the degree of narrowing, number of structures involved, and greater severity of these cases.

Furthermore, regarding lateral collapse, a study^(15)^ demonstrated that CPAP has a greater effect on the pharyngeal lateral walls than in any other upper airway obstruction site. The authors proposed DISE as a tool for early assessment of patients who have failed to adapt to CPAP, considering that it can help optimize CPAP by identifying patterns that suggest the need for variable pressures, as well as the identification of anatomical factors that need to be corrected^(15)^. In cases where anatomical factors associated with the musculature of the pharyngeal lateral walls are found, speech-language therapy^(7)^ may help to facilitate this process.

Conversely, one of the causes of non-adherence to CPAP refers to the collapse in the epiglottic site, in which higher pressure of the equipment increases the fall of the epiglottis in the anteroposterior direction^(13)^. Studies using OMT should be carried out to investigate potential assistance in these cases.

A frequent site of VAS collapse detected is at the base of the tongue^(10)^, which is often associated with mouth breathing during sleep, when muscle relaxation allows the tongue to move backward, blocking the airway. Based on this, OSA-specific OMT aims to favor the organization of respiratory function, mobilize and strengthen the nasopharynx and oropharynx muscles, especially the dilator muscles, in order to reduce upper airway collapse during sleep^(6,7,11)^. As for the eligibility prerequisites for this treatment and the disease etiology, specific OMT can be performed alone or associated with other treatments, being considered an emerging and promising therapy for SRD^(6,7,11)^. We must consider that OMT studies are relatively recent, as the first publication of a controlled and randomized study dates back to 2009^(6)^. Therefore, increasing knowledge of the obstructive site of OSA and its impact on pathophysiology is essential for health professionals and further research. Thus, this study provides information that can help professionals working in the area with additional analyses on the meaning of the locations and types of collapse detected, offering potential improvements for orofacial myofunctional therapeutic planning based on a better understanding of the pathophysiology of OSA and snoring.

In this sense, further therapeutic and randomized studies should be carried out to deepen the understanding of what can be achieved with the performance of oropharyngeal exercises according to the sites and types of collapse presented, making OMT more objective and individualized for each patient.

## FINAL COMMENTS

The obstructive sites described during DISE and illustrated through the analysis performed in this study are compatible with those already demonstrated in the literature, aiming to objectively direct therapy according to the pathophysiology of OSA. Understanding how each location and type of collapse affects the effectiveness of a treatment option also has an impact on determining eligibility for speech-language therapy (OMT). Understanding the sites of obstruction and types of collapse, as presented in this study, can be an important predictor of responses to orofacial myofunctional treatment, helping to understand the limitations of this therapeutic technique and directing the types of exercises proposed for each patient, customizing the OMT.
