# Supplementary material for: Obstructive Sleep Apnea: characterization of the obstructive site and type of collapse
Source: Codas. 2022 May 16;34(5):e20210208. doi: 10.1590/2317-1782/20212021208 (PMC9886182; doi:10.1590/2317-1782/20212021208)
Supplement: Translation

# Translation: Apneia Obstrutiva do Sono: caracterização do sítio obstrutivo e tipo de colapso

**Authors:** Andresa Santos da Silva, Fabio Augusto Winckler Rabelo, Eric Thuler, Fabiane Kayamori, Esther Mandelbaum Gonçalves Bianchini

**Affiliations:** 1 Programa de Pós-graduação em Fonoaudiologia, Pontifícia Universidade Católica de São Paulo – PUCSP - São Paulo (SP), Brasil.; 2 Departamento de Otorrinolaringologia, Hospital Samaritano - São Paulo (SP), Brasil.; 3 Departamento de Otorrinolaringologia, Hospital Sírio Libanês – HSL - São Paulo (SP), Brasil.

**Keywords:** Apneia Obstrutiva do Sono, Ronco, Faringe, Endoscopia, Terapia Miofuncional, Fonoaudiologia

## Abstract

A Apneia Obstrutiva do Sono caracteriza-se por episódios recorrentes de colapso parcial ou completo da faringe, seguidos de diminuição da saturação de oxihemoglobina e despertares frequentes. É considerada problema de saúde pública com importantes sintomas noturnos e diurnos, impactando qualidade de vida. Seus efeitos associam-se as áreas de competência da Fonoaudiologia. Para estabelecer diagnóstico e métodos de tratamento eficientes, profissionais devem conhecer a patogênese da obstrução da via aérea superior durante o sono. Visando contribuir para a compreensão da fisiopatologia da apneia obstrutiva do sono, elegibilidade de procedimentos terapêuticos individualizados e direcionamento para terapêutica miofuncional orofacial, o presente estudo tem como objetivo descrever e ilustrar os locais e tipos de colapso da via aérea superior durante o sono. Após processos éticos, foram analisados registros originais das sonoendoscopias de uma série de casos com diagnóstico polissonográfico de apneia obstrutiva do sono. As imagens das gravações dos exames foram analisadas por cinco profissionais com expertise na área do sono. Os locais obstrutivos e tipos de colapso foram apresentados conforme classificação vigente. Os vídeos foram divididos em capturas de tela, originando figuras de cada sítio anatômico: sem colapso e com colapso. Os resultados foram apresentados por imagens dos casos, que ilustram cada colapso, predominando colapso velofaríngeo: anteroposterior, lateral ou concêntrico; seguido por colapso orofaríngeo lateral; colapso anteroposterior na hipofaringe e colapso anteroposterior da epiglote. O entendimento dos locais de obstrução e tipos de colapso ilustrados nesse estudo pode ser um preditor de respostas terapêuticas, auxiliando a compreensão das limitações ou direcionando propostas para cada paciente.

## INTRODUÇÃO

A Apneia Obstrutiva do Sono (AOS) caracteriza-se por episódios recorrentes de colapso parcial ou completo da faringe, ocasionando eventos de hipopneia ou apneia, respectivamente, podendo ser seguidos de diminuição da saturação de oxihemoglobina e/ou despertares frequentes. É considerada um problema de saúde pública com importantes sintomas noturnos e diurnos, que impactam na qualidade de vida^(1)^. Os sintomas comuns são sonolência diurna excessiva; falta de atenção e memória; mudança de humor; aumento do risco de acidentes e doenças cardiometabólicas de longo prazo^(1)^. Os impactos dos distúrbios respiratórios do sono (DRS), quando não tratados, também podem ter efeitos como déficits neurocomportamentais e cognitivos, bem como modificações na qualidade da voz, da deglutição, comportamento auditivo e fluência da fala interferindo em diferentes níveis de comunicação ao longo da infância, adolescência, idade adulta e senilidade^(1)^. Esses efeitos estão fortemente associados as áreas de competência da Fonoaudiologia.

A patogênese da AOS é multifatorial e associa-se a fatores anatômicos e neuromusculares. Em relação aos fatores anatômicos, estudos têm mostrado^(2,3)^ o papel das estruturas craniofaciais, como deficiência maxilar, palato duro alto e estreito e mandíbula reduzida. Dentre os fatores neuromusculares destacam-se: faringe estreita ou colapsável e tecidos moles hipertrofiados, como tonsilas palatinas e pilares faríngeos aumentados, assim como tamanho, formato, volume do palato mole e tonsilas linguais. O envelhecimento e a obesidade também são considerados fatores contribuintes, respectivamente, para redução do tônus e da permeabilidade da Via Aérea Superior (VAS). A redução do tônus muscular e hipomobilidade contribuem para aumentar o volume e a flacidez da estrutura do tecido mole, que podem levar ao colapso da VAS, microdespertares e fragmentação do sono^(4)^.

O tratamento padrão para AOS é o uso de aparelho de pressão aérea positiva (PAP) durante o sono^(2)^; entretanto, a baixa adesão a esse tratamento compromete sua eficácia. O uso de dispositivos de avanço mandibular (DAM), com melhor aceitação, também é uma terapia clínica eficaz, visando o aumento do diâmetro da VAS com avanço mandibular titulável^(3)^. O tratamento cirúrgico para AOS inclui procedimentos de partes moles da orofaringe e da base da língua, assim como cirurgia esquelética, onde a expansão maxilar e o avanço maxilomandibular são também opções eficazes^(5)^, embora invasivas.

A Terapia Miofuncional Orofacial (TMO) é apresentada como uma alternativa e /ou opção de tratamento clínico complementar para os distúrbios respiratórios do sono (DRS). A TMO se propõe a realizar alterações da musculatura orofaríngea e funcionais, por meio de exercícios principalmente isotônicos e isométricos com os músculos orofaciais e orofaríngeos, além da organização funcional orofaríngea^(6)^. Esse conjunto de técnicas e procedimentos vem apresentando resultados satisfatórios no tratamento da AOS, com melhora significativa dos sintomas iniciais e melhor qualidade de vida^(7)^. No entanto, nem todos os indivíduos com AOS são elegíveis para TMO^(7,8)^ e definir os diagnósticos corretos para a seleção do paciente é crucial.

Na investigação diagnóstica dos distúrbios do sono (DS), a polissonografia tipo 1 (PSG1) é padrão ouro e consiste no registro simultâneo de variáveis fisiológicas durante o sono que apontam a distribuição dos estágios do sono, ocorrência e caracterização de eventos respiratórios centrais e/ou obstrutivos, monitoramento de gases sanguíneos como saturação de oxigênio e concentração de dióxido de carbono, além de eletromiograma e sensores de movimentos diferenciando várias interferências que podem definir microdespertares e consequente má qualidade do sono. A PSG1 é valiosa no diagnóstico dos DS de forma geral, uma vez que seus sintomas podem relacionar-se não apenas a ocorrência dos DRS. É uma avaliação também decisiva para detecção, caracterização e gravidade da AOS, entretanto não identifica o sítio obstrutivo e tipo de colapso^(8)^.

A determinação do sítio obstrutivo da VAS e o padrão de colapso têm se mostrado decisivos na compreensão do problema e orientação de seu tratamento, seja cirúrgico ou clínico^(9)^. A endoscopia do sono induzida por drogas (DISE) é uma avaliação endoscópica da VAS durante o sono induzido por medicação. Consiste em uma visualização tridimensional da VAS que permite a avaliação em tempo real dos locais obstrutivos em condições dinâmicas^(9,10)^. Estudos vêm demonstrando que os locais de obstrução e tipos de colapso podem definir os resultados dos diferentes tratamentos, sugerindo que a detecção do sítio obstrutivo e a compreensão dos mecanismos que propiciam a sua ocorrência têm impacto na decisão terapêutica^(8-11)^.

Considerando que a caracterização do sítio obstrutivo e compreensão do padrão de colapso dos pacientes com DRS propiciam direcionamentos específicos e individualizados visando a promoção de resultados mais promissores, é primordial que esses dados sejam conhecidos pelos fonoaudiólogos, buscando-se procedimentos da TMO específicos para as regiões e tipos de colapso.

Dessa forma, visando contribuir para a compreensão da fisiopatologia da AOS, elegibilidade de procedimentos terapêuticos individualizados e direcionamento para terapêutica miofuncional orofacial, o presente estudo tem como objetivo descrever e ilustrar os locais e tipos de colapso da VAS durante o sono de acordo com dados da DISE.

## APRESENTAÇÃO DOS CASOS CLÍNICOS

Trata-se de um estudo de casos exploratório-descritivo, aprovado pelo Comitê de Ética e Pesquisa da Pontifícia Universidade Católica de São Paulo (PUC-SP) sob o número 1.964.298 e CAEE 63408516.8.0000.5482.

Após os devidos procedimentos éticos, foram coletados os dados de 25 pacientes consecutivos do ambulatório de Otorrinolaringologia do Hospital Samaritano de São Paulo, com diagnóstico polissonográfico de AOS, e indicação de DISE, para definição terapêutica. Trata-se de avaliação eletiva, definida e realizada pelo ORL responsável pelo caso. Os exames foram realizados no centro de endoscopia utilizando nasofibrolaringoscópio flexível Olympus® / Scad® 3,4mm com supervisão de anestesiologista sob infusão alvo controlada do sono induzido por propofol, conforme protocolo previamente descrito^(12)^ capaz de reproduzir o mesmo Índice de Apneia e Hipopneia (IAH) do que o sono natural. Os parâmetros cardiorrespiratórios foram monitorados, e todos os equipamentos de segurança estavam disponíveis para atender a qualquer possível emergência. Todos os exames foram monitorados por fonoaudióloga com formação na área sono e as imagens gravadas digitalmente para posterior análise.

O processo de seleção dos vídeos do exame DISE obedeceu a um critério de inclusão para a seleção das imagens permitindo a visualização de cada local possível e tipos de colapso apresentados.

Foram excluídos os pacientes com índice de massa corpórea (IMC) >29.9, com comorbidades, assim como aqueles cujas amostras apresentassem problema técnico comprometendo a qualidade do vídeo.

A partir desses critérios, foram selecionados 11 casos com média de idade de 41 anos, mínima de 28 anos e máxima de 45; média do IMC de 25,05, mínimo de 23,4 e máximo de 27,3; todos do sexo masculino com AOS moderada, que retratam os achados do presente estudo.

Os participantes deste estudo faziam parte de uma pesquisa maior e assinaram o Termo de Consentimento Livre e Esclarecido, consentindo, desta forma, com a divulgação de seus resultados.

### Sítios e tipos de colapso da VAS

Os sítios obstrutivos da VAS foram descritos de acordo com a classificação “VOTE”^(9,10)^: (V) velofaríngeo, envolvendo palato mole, úvula e tecido da parede lateral da rinofaringe; (O) orofaríngeo, envolve tecidos moles da parede lateral da orofaringe e tonsilas palatinas; (T) lingual, envolve base da língua e parede posterior da orofaringe; (E) epiglótico, envolve a cartilagem epiglótica, dobra ari-epiglótica e área supra glótica que pode colapsar devido à redução da rigidez estrutural da cartilagem ou seu deslocamento posterior contra a parede posterior da faringe. Cada um dos sítios é também classificado quanto aos seus respectivos tipos de colapso: anteroposterior, lateral ou concêntrico. Os graus de obstrução são classificados como: (0) sem obstrução considerável <50%, nem vibração; (1) obstrução parcial (50 a 75%) com vibração; (2) obstrução completa (> 75%); e (x) não visível.

As imagens foram selecionadas e analisadas pelos autores deste estudo, dois otorrinolaringologistas e três fonoaudiólogas com expertise na área do sono, de forma separada seguida de confirmação conjunta, de acordo com o local e tipo de colapso apresentado.

Para facilitar a apresentação e visualização dos locais de colapso, assim como a análise de cada caso, foram feitas capturas de tela em dois ou três momentos de cada local: sem colapso, no momento de transição e no colapso. Entretanto, nem todos os casos foram ilustrados com imagens do momento de transição em função da rapidez da ocorrência do colapso, uma vez que essa é variável. Os casos em que a velocidade do colapso permitiu capturar o momento de transição foram apresentados com a imagem intermediária.

O grau de obstrução selecionado foi preferencialmente completo ou parcial sendo que as capturas de tela não retratam a vibração, pois exigem imagens dinâmicas, já que essa só é vista em vídeo. Embora as imagens tenham sido transformadas em fotos para permitir a publicação, os registros de vídeo estão disponíveis.

O Quadro 1 ilustra a forma como as imagens foram visualizadas e interpretadas, representando a visão do nasofibrolaringoscópio posicionado respectivamente na velofaringe e orofaringe, sem colapso.

**Quadro 1:** Exemplo dos dois posicionamentos do nasofibroscópio para análise das imagens obtidas: velofaringe e orofaringe

Imagens nasofibroscópicas em repouso, com o sujeito em vigília		
Nasofibroscópio posicionado na velofaringe		Sujeito acordado, deitado, em respiração nasal.
Nasofibroscópio posicionado na orofaringe		Sujeito acordado, deitado, em respiração nasal.

A Tabela 1 apresenta os dados referentes ao IMC, local e tipo de colapso de cada um dos pacientes analisados. Observou-se maior número de colapso velofaríngeo (cinco casos), seguido por colapso lingual (três casos), colapso orofaríngeo (dois casos) e colapso epiglótico (dois casos). Nota-se que apenas um caso apresentou dois tipos de colapso: lingual e epiglótico completos.

**Tabela 1:** Dados e achados dos sujeitos participantes

**Sujeitos**	**IMC**	**Local do colapso**	**Tipo de colapso**
1	23,4	V: Sítio Velofaríngeo	Anteroposterior completo
2	25,2	V: Sítio Velofaríngeo	Anteroposterior completo
3	24,3	V: Sítio Velofaríngeo	Circunferencial completo
4	27,3	V: Sítio Velofaríngeo	Lateral completo
5	24,8	V: Sítio Velofaríngeo	Circunferencial completo
6	24,5	O: Sítio Orofaríngeo	Lateral completo
7	26,2	O: Sítio Orofaríngeo	Lateral parcial
8	26,3	T: Sítio Lingual	Anteroposterior completo na hipofaringe com componente tonsilar lingual
9	25,4	T: Sítio Lingual E: Sítio Epiglótico	Anteroposterior completo na hipofaringe com componente tonsilar mínimo Anteroposterior completo
10	24,5	T: Sítio Lingual	Anteroposterior completo na hipofaringe sem componente tonsilar lingual
11	23,7	E: Sítio Epiglótico	Anteroposterior completo sem componente tonsilar lingual

As ilustrações dos casos foram apresentadas de acordo com o local e tipo de colapso, a seguir descritos.

- Sítio velofaríngeo

O sítio velofaríngeo pode apresentar três tipos de colapso: anteroposterior, lateral ou circunferencial (concêntrico), ilustrados nas Figuras 1 e 2.

**Figura 1:** Sítio Velofaríngeo (V). Imagens “A” sem colapso; imagens “B” durante colapso

**Legenda:** Sujeito 1: Imagem A1 sem colapso, B1 com colapso anteroposterior completo na velofaringe

**Legenda:** Sujeito 2: Imagem A2 sem colapso, B2 com colapso anteroposterior completo na velofaringe

**Legenda:** Sujeito 3: Imagem A3 sem colapso, B3 com colapso circunferencial completo na velofaringe

**Figura 2:** Sítio Velofaríngeo (V). Imagens “A” sem colapso; imagens ‘B” transição para o colapso com constrições parciais, imagens “C” durante colapso

**Legenda:** Sujeito 4: Imagem A4 sem colapso, B4 transição para o colapso lateral com constrições parciais, C4 colapso lateral completo na velofaringe

**Legenda:** Sujeito 5: Imagem A5 sem colapso, B5 transição para o colapso circunferencial com constrições parciais, C5 colapso circunferencial completo na velofaringe

O colapso anteroposterior corresponde à constrição do palato mole, úvula e parede posterior da velofaringe, estão representados na Figura 1. O colapso concêntrico ou circunferencial corresponde à constrição simultânea do palato mole, úvula, parede lateral e parede posterior da velofaringe, ilustrado na Figura 1 e na Figura 2. O colapso lateral corresponde à constrição das paredes laterais da rinofaringe, ilustrado na Figura 2.

Os sujeitos 1 e 2 apresentaram colapso velofaríngeo anteroposterior completo (Figura 1). Respectivamente A1 e A2 ilustram o local velofaríngeo sem colapso, B1 e B2 exemplificam o colapso anteroposterior completo.

O sujeito 4 apresentou colapso velofaríngeo lateral completo (Figura 2). Ilustrado por capturas em três momentos diferentes, mostrando, respectivamente, A4 local velofaríngeo sem colapso, B4 durante transição para o colapso e C4 com colapso lateral completo.

Os sujeitos 3 e 5 apresentaram colapso velofaríngeo circunferencial completo. As imagens do sujeito 3 (Figura 1) representam dois momentos distintos: A3, mostrando o sítio velofaríngeo sem colapso e B3 colapso circunferencial completo na velofaringe. As imagens do sujeito 5 (Figura 2) foram capturadas em três momentos, A5 sem colapso, B5 durante transição para o colapso e C5 ao ocorrer o colapso circunferencial completo.

- Sítio orofaríngeo

O sítio orofaríngeo pode apresentar um tipo de colapso: lateral, que ocorre em decorrência da constrição das paredes laterais da orofaringe, e depende não só do tamanho das tonsilas palatinas, mas também do tecido mole redundante nesta área, conforme Figura 3, ilustrado pelas capturas dos sujeitos 6 e 7.

**Figura 3:** Sítio Orofaríngeo (O). Imagens “A” sem colapso; imagens “B” com colapso

**Legenda:** Sujeito 6: Imagem A6 sem colapso, B6 com colapso completo das paredes laterais da orofaringe

**Legenda:** Sujeito 7: Imagem A7 sem colapso, B7 com colapso parcial das paredes laterais da orofaringe

As imagens foram capturadas em dois momentos diferentes, sem colapso A6 e A7 e ao ocorrer o colapso da parede lateral da orofaringe B6 e B7 (Figura 3). Vale ressaltar que ambos os sujeitos apresentaram o colapso sem existir tonsilas palatinas aumentadas.

- Sítio lingual (Hipofaringe)

O sítio lingual pode apresentar um tipo de colapso: anteroposterior, em decorrência da constrição entre a base da língua e a parede posterior da hipofaringe, conforme Figura 4, ilustrado pelas capturas dos exames dos sujeitos 8, 9 e 10.

**Figura 4:** Sítio da base da língua (T). Imagens “A” sem colapso; imagens “B” com colapso

**Legenda:** Sujeito 8: Imagem A8 base da língua com hipertrofia da tonsila lingual sem colapso em hipofaringe; B8 com colapso anteroposterior completo na hipofaringe com componente tonsilar

**Legenda:** Sujeito 9: Imagem A9 base da língua com mínima hipertrofia da tonsila lingual sem colapso na hipofaringe; B9 com colapso anteroposterior completo na hipofaringe com componente tonsilar mínimo

**Legenda:** Sujeito 10: Imagem A10 base da língua sem hipertrofia da tonsila lingual e sem colapso em hipofaringe; B10 com colapso anteroposterior completo na hipofaringe sem componente tonsilar

O sujeito 8 apresenta hipertrofia da tonsila lingual e o sujeito 9 apresenta mínima hipertrofia unilateral dessa tonsila. As capturas A8 e A9 mostram a região da hipofaringe sem colapso para facilitar a comparação com as imagens B8 e B9, que definem o colapso anteroposterior da hipofaringe com componente tonsilar lingual.

O sujeito 10 (A10 e B10) apresentou colapso anteroposterior da base da língua sem qualquer hipertrofia das tonsilas linguais. A captura A10 mostra este local sem colapso e B10 mostra o colapso anteroposterior completo na hipofaringe causado pelo deslocamento posterior da base da língua após o relaxamento muscular. Vale ressaltar que os três casos apresentaram colapso completo, independente do volume da tonsila lingual. Entretanto, esse é considerado fator agravante a esse tipo de colapso.

- Sitio Epiglótico

Em relação ao sítio da epiglote, podem ser encontrados dois tipos de colapso: anteroposterior e lateral. Na presente amostra, foram encontrados dois casos de colapso epiglótico, sujeito 9 e sujeito 11, ambos do tipo anteroposterior, como consequência do deslocamento posterior da epiglote contra a parede posterior da hipofaringe, conforme ilustrado pela Figura 5.

**Figura 5:** Sítio Epiglótico (E). Imagens “A” sem colapso; imagens “B” com colapso

**Legenda:** Sujeito 09: Imagem A11 local epiglótico sem colapso, B11 colapso anteroposterior completo causado pelo deslocamento posterior da epiglote contra a parede hipofaríngea posterior com componente tonsilar lingual mínimo

**Legenda:** Sujeito 11: Imagem A1:sem colapso, B12 com colapso anteroposterior completo causado pelo deslocamento posterior da epiglote contra a parede hipofaríngea posterior, sem componente tonsilar lingual

As capturas A11 e A12 demonstram o sítio da epiglote sem colapso, facilitando a comparação com as imagens B11 e B12, que demonstram o colapso anteroposterior completo da epiglote. Vale ressaltar que o sujeito 9 apresentou dois tipos de colapso em momentos distintos da avaliação DISE: lingual (Figura 4) e epiglótico (Figura 5), ambos anteroposterior, sugerindo componente muscular de queda da língua.

No presente estudo não foi encontrado nenhum caso que possibilitasse ilustrar o colapso lateral do sítio da epiglote, sendo esse raro envolvendo instabilidade da rigidez da epiglote lateralmente^(10,13)^.

Em relação as limitações do estudo, por tratar-se de avaliação eletiva e de alto custo, destacam-se o número de casos analisados que, embora tenham permitido apresentar quase todos os tipos de colapso com exceção do epiglótico lateral, não permitem generalizar a frequência de ocorrência de cada sítio e tipo de colapso. A ausência de literatura científica na área da Fonoaudiologia sobre o tema abordado até o presente momento, também impossibilita comparações com pesquisas anteriores e associações com consequente discussão mais ampla sobre os dados apresentados.

## DISCUSSÃO

No presente estudo, ao apresentar uma série de 11 casos, visando descrever e ilustrar os locais e tipos de colapso da VAS durante o sono e contribuir para a compreensão da fisiopatologia da AOS, foi encontrado maior número de casos com colapso no sítio velofaríngeo, seguido por colapso orofaríngeo, colapso lingual, enquanto o colapso epiglótico foi o menos frequente. Esses dados corroboram com estudos anteriores que demonstram a região velofaríngea como o local mais comum de colapso da VAS durante o sono^(10,12,14)^. Entretanto, por tratar-se de série de casos, os dados não permitem generalizar a frequência de ocorrência de cada sítio e tipo de colapso, assim como o grau do de obstrução.

A AOS tem sido amplamente estudada nas últimas décadas, diversos estudos trouxeram suas contribuições para melhor compreensão desta doença, incluindo seus sintomas, definição diagnóstica e abordagem terapêutica. Embora o uso de dispositivos de pressão positiva nas vias aéreas (PAP) tenham obtido bons resultados a baixa adesão compromete sua eficácia, o que justifica a busca por opções alternativas de tratamento^(2,3,5-7,11)^.

Considerando a atuação fonoaudiológica nos DRS^(6,7)^ há evidências que demonstram que a TMO, com exercícios orofaríngeos e reorganização funcional, consiste em uma opção de tratamento eficaz para muitos pacientes. O foco desta terapia é melhorar a competência muscular, flexibilidade e tônus da região orofaríngea. Assim, é importante buscar instrumentos que possam otimizar o entendimento da fisiologia e fisiopatologia do sono, visando otimizar as definições para elegibilidade da TMO no tratamento dos DRS^(6,7,11)^.

A DISE tem se caracterizado como uma importante ferramenta na detecção de sítios obstrutivos da VAS, auxiliando na seleção do paciente quanto a definição terapêutica clínica e/ou cirúrgica e seus resultados^(9,10,14)^. No que se refere a conduta clínica voltada para a TMO a partir da caracterização do sítio obstrutivo e tipo de colapso, podem ser direcionadas possíveis considerações terapêuticas. A seleção dos exercícios poderá ser voltada para aqueles que atuam diretamente na região em que ocorre o colapso, uma vez que se refere a maior debilidade na musculatura. Tal direcionamento clínico contribui para elaboração de planejamento terapêutico individualizado com foco na musculatura acometida, nem sempre constatada em avaliação clínica. Com a possibilidade de redução do número de exercícios propostos pode-se obter melhor adesão ao tratamento miofuncional orofacial. A compreensão do colapso também poderá considerar uma terapêutica que busque ir contra esses colapsos, isto é, evitar reproduzir a obstrução com determinado exercício e priorizar aqueles que gerem abertura do espaço, além do fortalecimento da estrutura acometida. Os exercícios a serem indicados podem ser aqueles que não propiciam o movimento de fechamento/obstrução. A partir dessas definições novos estudos controlados e randomizados devem ser conduzidos para verificação de resultados terapêuticos.

Comparando os tipos de colapso, um estudo de coorte^(10)^ refere que os pacientes que apresentaram colapso circunferencial completo na velofaringe eram significativamente mais propensos a ter Índice de Apneia e Hipopneia (IAH) e IMC mais elevados, quando comparados àqueles que apresentavam colapso anteroposterior no mesmo local obstrutivo, que apresentavam IMC significativamente menor. Na presente série de casos esses dados não foram decisivos, uma vez que o IMC e o grau de gravidade da AOS eram semelhantes. Vale ressaltar que estudos apontam o colapso circunferencial na velofaringe como mais difícil de tratar do que outros locais e padrões de colapso, o que o caracteriza um estreitamento anatômico mais severo^(10,14)^. A abordagem mecânica, utilizando um dispositivo de pressão positiva contínua, ou mesmo aparelho intra-oral, é apontada como a mais adequada nestes casos^(2,3)^. Portanto, a detecção do colapso circunferencial completo durante a DISE mostrou importante direcionamento terapêutico^(14,15)^. Nesse sentido, talvez a abordagem com TMO não seja suficiente para tratar esse tipo de colapso, ou necessite de maior definição na escolha dos exercícios em função do grau de estreitamento, número de estruturas envolvidas e maior gravidade desses casos.

Também quanto ao colapso lateral, estudo^(15)^ demonstrou que o CPAP tem um efeito maior nas paredes laterais da faringe do que em qualquer outro local obstrutivo da VAS. Os autores propuseram a DISE como uma ferramenta para avaliação precoce de pacientes que não conseguiram se adaptar ao CPAP, considerando que pode ajudar a otimizar o CPAP pela identificação de padrões sugiram a necessidade de pressões variáveis, bem como a identificação de fatores anatômicos que precisam ser corrigidos^(15)^. Nos casos em que forem constatados fatores anatômicos associados com a musculatura das paredes laterais da faringe, a atuação fonoaudiológica^(7)^ pode auxiliar na facilitação desse processo.

Por outro lado, uma das causas de não adesão ao CPAP refere-se ao colapso no sítio epiglótico, no qual o aumento da pressão do equipamento aumenta a queda da epiglote no sentido anteroposterior^(13)^. Estudos com utilização da TMO parecem necessários para verificação de possível ajuda nesses casos.

Um local frequente de colapso de VAS detectado é na base da língua^(10)^, que muitas vezes se associa à respiração oral durante o sono, quando o relaxamento muscular permite que a língua se mova para trás, bloqueando a via aérea. Com base nisso, a TMO específica para AOS tem como objetivo favorecer a organização da função respiratória, mobilizar e fortalecer os músculos da nasofaringe e orofaringe, principalmente os músculos dilatadores, visando reduzir o colapso da VAS durante o sono^(6,7,11)^. Respeitando os pré-requisitos de elegibilidade para este tratamento e a etiologia da doença, a TMO específica pode ser realizada isoladamente ou associada a outros tratamentos, sendo considerada terapia emergente e promissora para os DRS^(6,7,11)^. Devemos considerar que os estudos de TMO são relativamente recentes, pois a primeira publicação de um estudo controlado e randomizado, data de 2009^(6)^. Portanto, aumentar o conhecimento do local obstrutivo da AOS e seu impacto na fisiopatologia é fundamental para esses profissionais e para futuras pesquisas. Dessa forma, o presente estudo fornece informações que podem auxiliar o profissional que atua na área, com análises adicionais sobre o significado dos locais e tipos de colapso detectados, oferecendo possíveis melhorias para o planejamento terapêutico miofuncional orofacial a partir de melhor compreensão da fisiopatologia da AOS e do ronco.

Nesse sentido, novos estudos terapêuticos e randomizados devem ser realizados para aprofundar a compreensão do que pode ser alcançado com a realização de exercícios orofaríngeos de acordo com os locais e tipos de colapso apresentados, tornando a TMO mais objetiva e individualizada para cada paciente.

## COMENTÁRIOS FINAIS

Os sítios obstrutivos descritos durante a DISE e ilustrados por meio da análise realizada neste estudo são compatíveis aqueles já demonstrados na literatura, visando direcionar objetivamente a terapia de acordo com a fisiopatologia da AOS. A compreensão de como cada local e tipo de colapso afeta a eficácia de uma opção de tratamento também tem impacto na definição da elegibilidade do tratamento fonoaudiológico. O entendimento dos locais de obstrução e tipos de colapso, conforme apresentado no presente estudo, pode ser um importante preditor de respostas ao tratamento miofuncional orofacial, auxiliando no entendimento das limitações desta técnica terapêutica e direcionando os tipos de exercícios propostos para cada paciente, customizando-se a TMO.
